# Detection of Low RCS Supersonic Flying Targets with a High-Resolution MMW Radar

**DOI:** 10.3390/s20113284

**Published:** 2020-06-09

**Authors:** Nezah Balal, Yael Balal, Yair Richter, Yosef Pinhasi

**Affiliations:** Faculty of Engineering, Ariel University, Ariel 40700, Israel; nezahb@ariel.ac.il (N.B.); yairrichter@gmail.com (Y.R.); yosip@ariel.ac.il (Y.P.)

**Keywords:** Doppler radar, millimeter wave radar, catalog targets

## Abstract

In this study, the detection of a low radar cross-section (RCS) target moving at a very high speed using a high-resolution millimeter-wave radar is presented. This real-time detection is based on the transmission of a continuous wave and heterodyning of the received signal reflected from the moving target. This type of detection enables one to extract the object’s movement characteristics, such as velocity and position, while in motion and also to extract its physical characteristics. In this paper, we describe the detection of a fired bullet using a radar operating at an extremely high-frequency band. This allowed us to employ a low sampling rate which enabled the use of inexpensive and straightforward equipment, including the use of small antennas that allow velocity detection at high resolution and with low atmospheric absorption.

## 1. Introduction

The real-time detection of small, high-speed objects, such as fired bullets, is essential for security and civilian applications [1,2,3,4]. This type of detection enables one to track and identify the sources of gunshots as well to determine the instantaneous velocity of objects, calculate the drag constant of objects, and analyze the ballistic movement of objects.

The devices currently available for this type of detection operate in the optical field, and thus require the use of an extensive array of sensors located at various points as well as synchronization between all these sensors [5,6,7,8]. When the detection location is known, it is possible to use high-speed photography. Using this method limits the number of images that can be taken per second. Therefore, the maximum speed of the object to be detected and the computational speed resolution of this system are limited.

Doppler radar-based systems have several advantages over optical systems for real-time detection. The Doppler frequency, which is directly linked to the relative speed between the moving object and the radar, can be measured at a relatively low sampling rate, and therefore, no unique or expensive equipment is required for such measurements. Measuring the instantaneous Doppler frequency enables one to calculate the instantaneous velocity of an object at a high resolution.

In this article, we propose a real-time detection system based on a millimeter-wave (MMW) Doppler radar that transmits a continuous wave (CW) waveform. The reflected wave, scattered from a moving target is shifted in frequency due to the Doppler effect. Millimeter wave (MMW) radars are becoming more and more commercial and applicable due to recent technological developments [9,10,11]. They are employed as detection measures in many applications, such as collision avoidance radars in automobiles [12,13]. Detecting a target with a Doppler MMW radar has several advantages compared to other remote sensing technologies operating in infrared or optical wavelengths, mainly in foggy conditions and stationary background clutter scenarios [14,15,16]. Although millimeter waves are attenuated by fog, haze, and rain [17], they still can be used for short-range detection, where optical sensors completely fail.

This study is aimed at the realization of tracking small moving targets using a high-resolution radar operating in millimeter wavelengths (W-band). Considerations are presented for an efficient detection of stealth, small radar cross-section (RCS) targets, demonstrating deviations in their instantaneous velocities even in super-sonic speeds. The instantaneous velocity of a high-speed moving object and its drag constant can both be determined using this simple and inexpensive system. An analysis, practical experiments, and their results are presented.

This paper is organized as follows: Section 2 describes the principles of the micro-Doppler radar operation. Section 3 describes the challenges in detecting a small, high-speed object. The experimental setup is presented in Section 4. In Section 5, we present two experiments that differ in terms of radar location. The time–frequency signal obtained from a fired bullet is shown, analyzed, and its object coefficient drag is extracted. Section 6 summarizes and concludes the paper.

## 2. Doppler Radar

**Figure 1:**
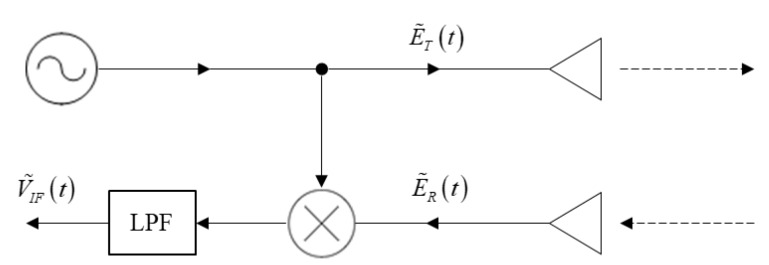
continuous wave Doppler radar.

The principle scheme of a continuous wave (CW) micro-Doppler radar is shown in Figure 1. The transmitted waveform is a millimeter wave carrier at a constant frequency $f_{0}$:

(1) $${\tilde{E}}_{T_{x}}\left(t\right)=A_{T_{x}}e^{j2\pi f_{0}t},$$

scattered by the target, the reflected signal received by the radar is

(2) $${\tilde{E}}_{R_{x}}\left(t\right)=A_{R_{x}}e^{-j\left[2k\cdot r\left(t\right)-\theta \right]}\cdot e^{j2\pi f_{0}t},$$

where $A_{T_{x}}$ and $A_{R_{x}}$ are the amplitudes of the transmitted and received signals, respectively, $k=2\pi f_{0}/c$ is the wavenumber (c is the speed of light), r(t) is the distance to the moving target (here multiplied by a factor of 2 because it contains the path to and from the radar and the target), and θ is a constant phase shift. The detection is based on a heterodyne mixing of the reflected signal (2) scattered from the target, with the transmitted CW carrier (1), resulting in the following product:

(3) $${\tilde{V}}_{IF}\left(t\right)={\tilde{E}}_{T_{x}}\left(t\right)\cdot {\tilde{E}}_{R_{x}}{}^{*}\left(t\right)=A_{T_{x}}A_{R_{x}}e^{j\left[2k\cdot r\left(t\right)-\theta \right]}.$$

An inspection of Equation (3) reveals a time-varying phase,

(4) φ(t)=2k⋅r(t)−θ,

from which the instantaneous Doppler frequency shift can be derived:

(5) $$f_{d}\left(t\right)=\frac{1}{2\pi }\frac{\partial \varphi \left(t\right)}{\partial t}=\frac{1}{2\pi }2k\cdot \dot{r}\left(t\right)=\frac{2f_{0}}{c}v_{r}\left(t\right),$$

where $v_{r}\left(t\right)=\dot{r}\left(t\right)$ is the radial target velocity related to the radar. In a scenario where the sensor observes the moving target at angle α, the radial velocity with respect to the radar is given by

(6) $$v_{r}\left(t\right)=v\left(t\right)\cdot \cos\alpha ,$$

where v(t) is the velocity of the target. The resulting Doppler instantaneous frequency shift $f_{d}\left(t\right)$ of the intermediate frequency (IF) signal obtained at the output of the mixer is proportional to the radial velocity of the target, including the deviations associated with the target velocity. It is important to note that according to Equation (5), the intermediate frequency is proportional to the carrier frequency $f_{0}$. Increasing $f_{0}$ results in a higher frequency deviation $f_{d}\left(t\right)$ at the IF. For example, a target moving at a super-sonic radial speed of $v_{r}=1200\;m/s$ will be detected by a millimeter wave radar operating at $f_{0}=94\;\mathrm{GHz}$ producing an IF signal with a frequency of $f_{d}=752\;\mathrm{kHz}$ at the mixer output. The IF signal can then be digitized using an analog-to-digital (A/D) converter with a sampling rate of few MHz for further processing.

## 3. Challenges in Detecting Small, High-Speed Objects

In the followings, a fired bullet is used to demonstrate the detection of super-sonic small RCS targets. Detection of such an object, requires special considerations due to the low signal-to-noise ratio (*SNR*) expected in the detector. The *SNR* in the radar receiver is given by

(7) $$SNR=G_{r}\frac{\lambda^{2}\sigma }{{\left(4\pi \right)}^{3}r^{4}}G_{t}P_{t}\frac{T_{I}}{N_{0}},$$

where $P_{t}$ is the transmitter power, and $G_{t}$ and $G_{r}$ are the antenna gains of the transmitter and receiver, respectively. λ is the transmitter wavelength ($\lambda =c/f_{0}$), r is the range from the transmitter to the target, and $N_{0}$ is the spectral power density of the noise. σ is the radar cross-section (RCS). The RCS value of an object depends on its physical shape, transmission wavelength, and transmission angle relative to the object. A bullet can be approximated as a ball shape. The RCS of a ball depends on the ratio between the transmission wavelength λ and the radius a of the ball. For a≫λ, where the ball’s radius is much higher than the transmission wavelength (termed as the ‘optical case’), the RCS is defined by $\sigma =\pi a^{2}$ [18,19]. In case of a tiny radius a, the resulted bullet’s RCS value is small, leading to a low *SNR* at the sensor.

Usually the integration time $T_{I}$ in Equation (7) is the target illumination time. Due to the high speed of the bullet, its time of flight is very short, limiting the signal to noise at the receiver. Moreover, in order to track the instantaneous velocity of the bullet along its flight path, it is necessary to divide the flying duration into temporal windows, during each of which a short time Fourier transformation (STFT) is carried out, as explained in the following.

Using Equation (5), the object instantaneous velocity resolution $\Delta v_{r}$ can be expressed via the frequency resolution $\Delta f_{d}$ of the STFT:

(8) $$\Delta v_{r}=\frac{c}{2f_{0}}\Delta f_{d}.$$

The relationship between the frequency resolution and the integration time is $\Delta f_{d}=1/T_{I}$. Therefore, Equation (8) can now be re-written to express the velocity resolution (8) in terms of the STFT integration time $T_{I}$

(9) $$\Delta v_{r}=\frac{c}{2f_{0}}\frac{1}{T_{I}}.$$

Inspection of Equation (8) reveals that as the carrier frequency $f_{0}$ is increased, higher speed resolution is obtained for a given integration time $T_{I}$. This demonstrates the advantage of utilizing extremely high frequencies, as millimeter waves for tracking velocities of fast-moving targets.

Particular attention is required when choosing the appropriate temporal width of the STFT window. The integration time $T_{I}$ should be long enough to allow a sufficient resolution speed, as given by (9), but not too long in order to enable following of the temporal changes in the instantaneous velocity of the target. It is important to note that transmission at high carrier frequency $f_{0}$ compensates for employing a narrower temporal window in the STFT, while maintaining the required velocity resolution. Shortening the integration time results in a decreased signal-to-noise ratio, as expressed by Equation (7). In order to assure efficient detection and velocity tracking in the experiments, a trade-off is made between SNR and velocity resolution in the selection of an optimal $T_{I}$. In the present study, the STFT integration was set to $T_{I}=0.25\;\mathrm{ms}$, resulting in a velocity resolution of $\Delta v_{r}=6.38\;m/s$, which is accurate enough, since the bullet speed is at least two orders of magnitude higher.

## 4. Experimental Setup

**Figure 2:**
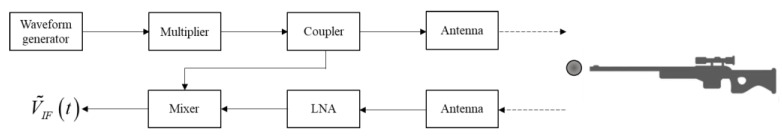
A scheme of the continuous wave micro-Doppler radar.

Figure 2 presents a scheme for the Doppler radar operating at millimeter wavelengths. The directivity of the antenna is crucial in many scenarios, especially in this particular application dealing with a small moving target that has a low RCS. Choosing a radar operating at an extremely high frequency band, 94 GHz, enables the realization of a directive antenna with a relatively small aperture. This 94 GHz frequency is within the atmospheric W-band transmission window and has the lowest atmospheric absorption. This enables us to increase the distance to the target, thereby enabling detection even in adverse weather conditions.

**Figure 3:**
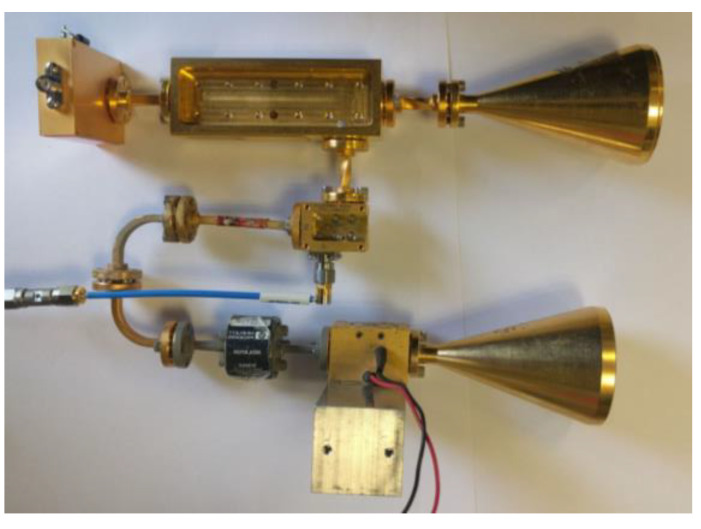
The continuous wave micro-Doppler radar.

The radar we used in this experiment is composed of an RF generator producing a continuous sine wave at 15.67 GHz. The wave frequency is multiplied six times to generate a 94 GHz carrier. A small value for the generated power (10 dB of the transmitted power) is coupled to the mixer in the receiving chain via a coupling port. Two identical horn lens antennas are employed for transmission and reception, each with a gain of 30 dBi (corresponding to a beam width of about 6.5°). The gain of the low noise amplifier (LNA) of the receiver is 30 dB, and its noise value is 5 dB. The product obtained at the mixer output lies at intermediate frequencies (IF) determined by the Doppler shifts of the signal reflected by the target; in our case, in frequencies of $f_{d}=313-752\;\mathrm{kHz}$. The IF signal is sampled by an analog-to-digital (A/D) converter at variable rates corresponding to the frequencies expected at the detector’s output. In our experiment, we used a sampling frequency of 20 MHz, which is well above the Nyquist limit. A photo of the radar is given in Figure 3.

## 5. Experimental Study

**Figure 4:**
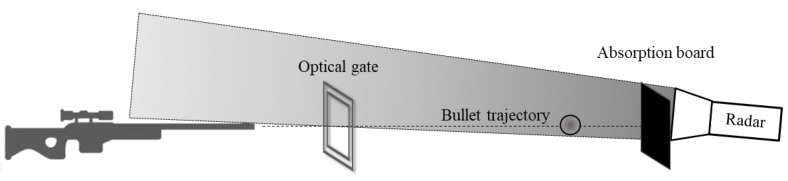
Illustration of the radar position; the radar is placed opposite to the gun.

In our experiments, we used a radar operating at an extremely high frequency band of 94 GHz. In a 25-m-long room, we placed a gun on one end and an absorption board at the opposite end, as shown in Figure 4. An optical gate system was placed within the flight path of the bullet. This system measured the instantaneous velocity of the bullet, which enabled verification of our measurement results.

**Figure 5:**
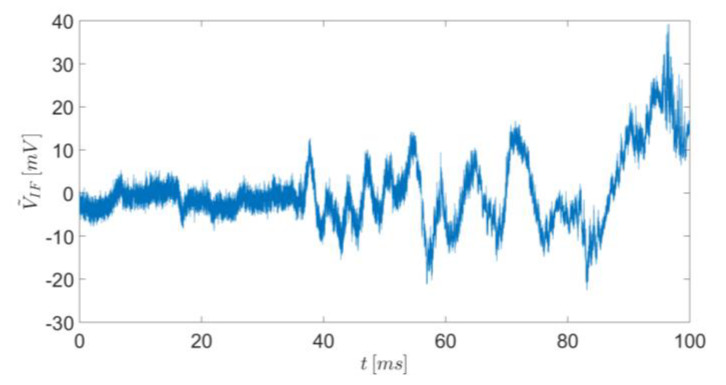
Measurement results of the intermediate frequency signal in the time domain.

In our first experiment, we placed the radar opposite to the gun and a few cm near the bullet’s flight trajectory so that the bullet moved towards the radar (see Figure 4). The fired bullet exits the gun barrel at a speed of about 1200 m/s, so that the flight time was about 20 ms along the shooting gallery. Figure 5 shows the time domain of the detected IF signal received by the radar.

In order to measure the instantaneous radial velocity of the fired bullet, a STFT was carried out on the above IF signal results at the Doppler instantaneous frequency. This frequency is connected to the instantaneous velocity by Equation (5). The STFT was performed for an integration temporal window of 0.25 ms. According to Equation (5), the speed resolution is 6.38 m/s, which is sufficient for tracking the bullet velocity along its flight path.

**Figure 6:**
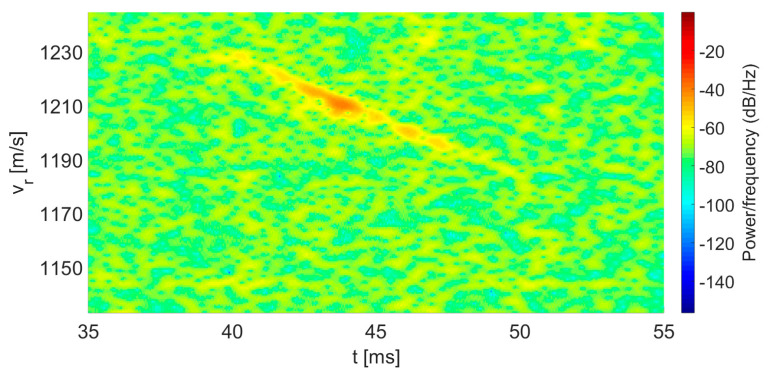
Spectrogram of the instantaneous bullet velocity vs. time.

**Figure 7:**
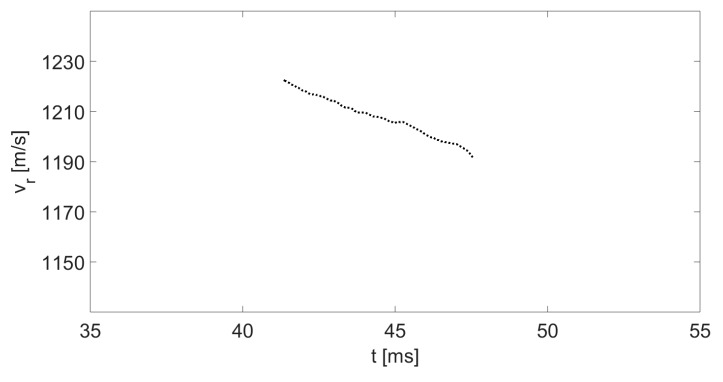
The instantaneous velocity of the fired bullet as a function of time.

In Figure 6, we present a three-dimensional spectrogram of the instantaneous velocity vs. time. The color of the graph represents the intensity of the IF signal at a particular frequency and time; blue corresponds to low levels, while red represents higher intensities. Thus, the red curve presents the peak of the instantaneous velocity of the bullet as a function of time. Figure 7 illustrates the graph of the bullet’s instantaneous velocity as a function of time by displaying the peaks of the spectrogram of Figure 6.

The results of the velocity measurements obtained from the radar and the optical gate are identical. The distance r between the moving target and the radar is related to its velocity $v_{r}$ by the correlation

(10) $$r=\int_{0}^{t}v_{r}\left(t\right)dt.$$

**Figure 8:**
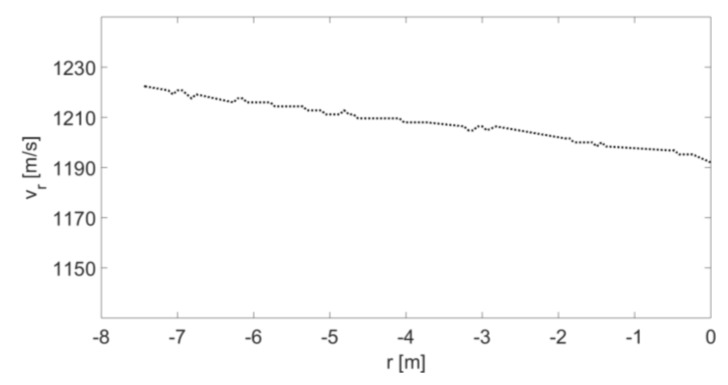
Instantaneous velocity of a small object as dependent on r.

By using (10), we can display the velocity $v_{r}$ (from Figure 7) as a function of r; the result is presented in Figure 8. The point at 0 m on the *x*-axis represents the location of the radar and the absorption board. As expected, the velocity of the object fades as it approaches the radar. We note that the radar field of view along the bullet's flight path is limited because the optical system blocks the first 8 meters of the bullet trajectory from the gun barrel. In addition, the gun and the absorption board are located about 2 meters from the edges of the room.

**Figure 9:**
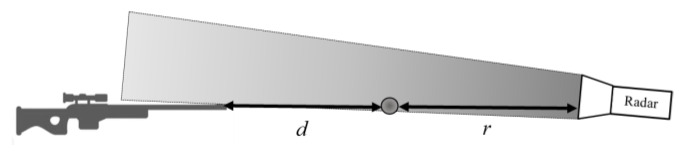
Illustration of the distance between the moving target to the radar r and the initial point d.

**Figure 10:**
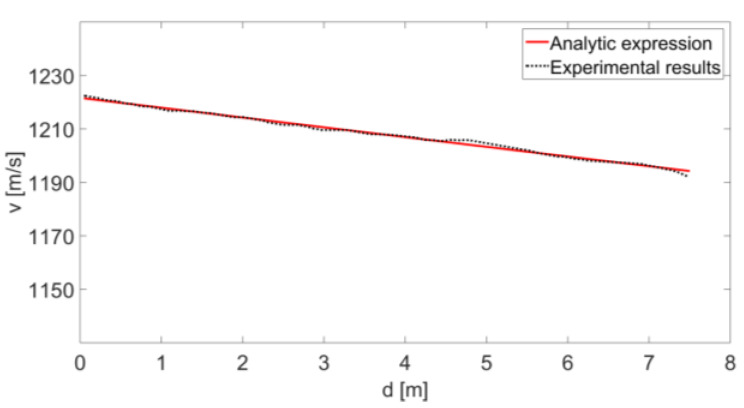
Instantaneous velocity of a small object as dependent on d.

In this configuration, where the radar is placed opposite to the gun, the angle α is approximately zero because the bullet’s flight trajectory and the radar are placed on the same axis (see Figure 9). Therefore, we assume that the velocity of the target and the radial velocity are equal; i.e., $v\left(d\right)≅v_{r}\left(r\right)$. Furthermore, the results of Figure 8 can be shown as v(d) (as a function of d; see “Experimental results” in Figure 10).

When an object moves in the middle, its velocity decreases in accordance with its drag constant. The relationship between the object speed v and the drag constant γ is given by [20,21,22]:

(11) $$v\left(d\right)=v_{0}e^{-\gamma \cdot d},$$

where d is the position of the object relative to the initial point, and $v_{0}$ is the initial speed. The drag constant is a function of the shape of the bullet and its mass. Therefore, when the speed of the object is known, depending on its position, the drag constant γ can be extracted from (11), and the object’s characteristics can be assessed. Using simulation software, we performed a regression of the “Experimental results” curve (in Figure 10) using Equation (9). This regression result is presented in Figure 10 as the “Analytic expression” and provides the value $\gamma =3.021{\;\mathrm{km}}^{-1}$, with an R-square of 0.9933, which shows how well the terms (data points) fit the curve.

The drag constant γ can be calculated using the following formula [21]:

(12) $$\gamma =\frac{\rho C_{d}\pi D^{2}}{8m},$$

where *ρ* is the air density ($1.225\;\mathrm{kg}/m^{3}$) at sea level, *D* is the bullet diameter (2 cm), *m* is the bullet mass (53 g), and the corresponding coefficient is $C_{d}=0.85$ [21]. Substituting these values in (12) leads to the theoretical value of $\gamma =3.086{\;\mathrm{km}}^{-1}$. The values of the drag constant obtained by the experimental results and the theoretical results show a high correlation. These results demonstrate that an object’s drag constant can be estimated for small objects through an analysis of their supersonic velocity.

**Figure 11:**
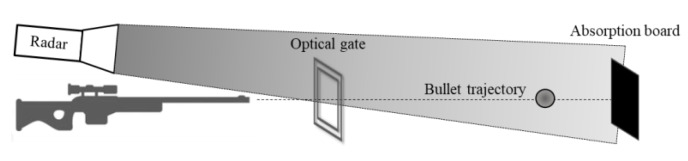
Illustration of the second experimental setup; the radar is placed behind the gun.

**Figure 12:**
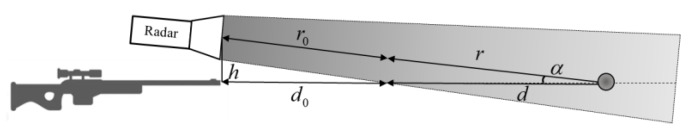
Illustration of the different parameters.

In our second experiment, we placed the radar behind the gun (see Figure 11) such that the bullet moved away from the radar. A bullet of a different shape was fired from the gun at a speed of about 500 m/s so that the flight time was about 50 ms. The angle α (see Figure 12) between the direction of the transmission and the bullet’s flight trajectory changed as a function of the bullet’s position by

(13) $$\cos\left[\alpha \left(t\right)\right]=\frac{d\left(t\right)+d_{0}}{r\left(t\right)+r_{0}},$$

where $d_{0}$ and d(t) describe the position of the bullet on its trajectory, and r(t) describes the distance from the radar to the moving bullet. $d_{0}$ and $r_{0}$ are the distances to the detection starting point of the bullet by radar, and h is the distance between the bullet’s initial flight trajectory and the radar.

**Figure 13:**
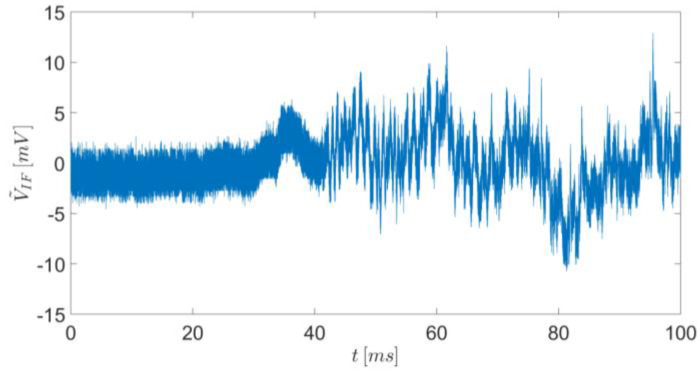
Measurement results: The intermediate frequency (IF) signal in the time domain.

**Figure 14:**
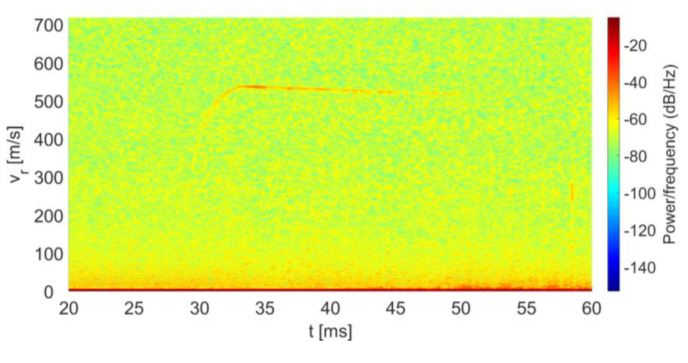
The instantaneous velocity of the fired bullet as a function of time.

**Figure 15:**
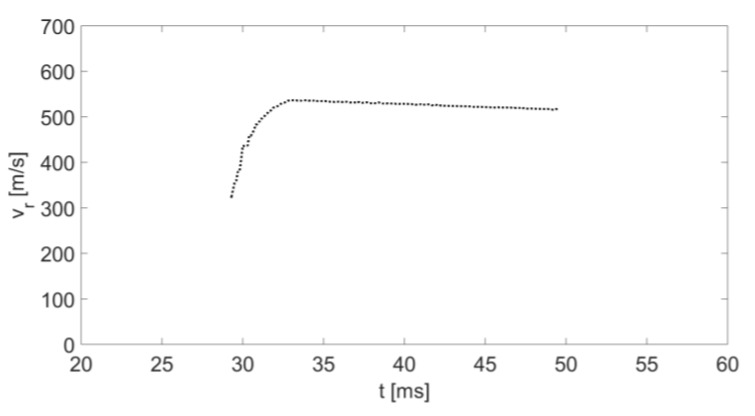
The instantaneous velocity of the fired bullet as a function of time.

**Figure 16:**
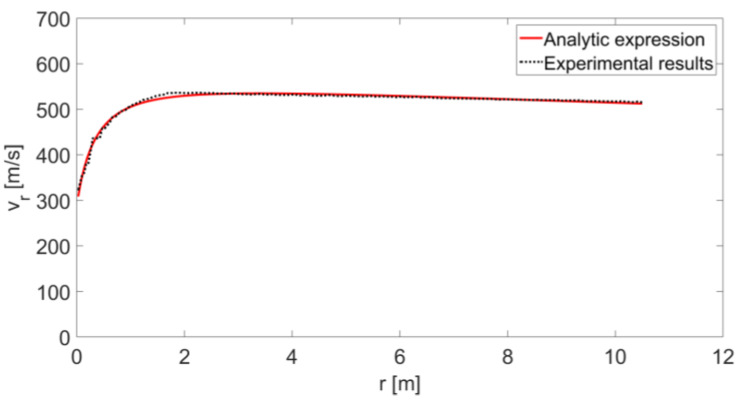
Instantaneous velocity of the small object as dependent on r.

Figure 13 shows the detected IF signal received by the radar when measuring the instantaneous speed of a fired bullet. Figure 14 shows the instantaneous radial velocity calculated by using STFT of the detected frequency. It can be seen that for a short time at the beginning of its movement, the radial speed increased steeply and then decreased. At the beginning of the movement, the value of angle α was large and rapidly reduced to an angle that approaches zero ($d_{0}+d\gg h$); i.e., the initial value of cosα was low and increased rapidly to 1, resulting in an increase in the radial velocity projection (see Equation (6)). The fade-in velocity was due to the bullet’s drag constant, as indicated by (11). Only the bullet’s speed is displayed in Figure 15, and by using (10), we obtained the “Experimental results” curve in Figure 16.

The velocity of the bullet measured by the optical gate was identical to the results obtained from the curve obtained by the radar.

By substituting Equations (11) and (13) into (6) and by using $d=\sqrt{{\left(r+r_{0}\right)}^{2}-h^{2}}-d_{0}$(the Pythagorean theorem), we can obtain the following equation:

(14) $$v_{r}\left(r\right)=v_{0}e^{-\left(\sqrt{{\left(r+r_{0}\right)}^{2}-h^{2}}\right)\cdot \gamma }\cdot \frac{\sqrt{{\left(r+r_{0}\right)}^{2}-h^{2}}}{r+r_{0}}.$$

Equation (14) presents an analytical expression of the relationship between the radial velocity measured by the radar and the distance r between the radar and the small moving object. This relationship is presented in Figure 16 by the “Experimental results” curve that was generated from the radar measurement results in the experiment. By regressing this curve to Equation (12), it is possible to estimate the distance h between the bullet’s flight trajectory and the radar and find the drag constant γ. The regression result is presented in Figure 16 as the “Analytic expression”, providing the values of $\gamma =8.1{\;\mathrm{km}}^{-1}$ as well as of h=0.8052 m (which fits the physical location of the radar in respect to the barrel). As expected, in this experiment, we observed a different value for the drag constant due to a differently shaped bullet.

These results show that by analyzing the velocity of a small object, its drag constant and parameter h can be estimated.

## 6. Summary and Conclusions

This paper presents a real-time detection technique of low RCS targets flying at supersonic speeds and identification of their movement and physical characteristics. A directive continuous wave MMW radar, operating in the W-band, was employed to detect frequency shifts due to the target’s movement. This was done by heterodyning the reflected signal with the transmitted one. Short time Fourier transformation (STFT) of the resulting IF signal generates a spectrogram of the instantaneous Doppler frequency shifts, corresponding to the temporal target velocity.

It is shown that utilization of millimeter wavelengths enables detection of low RCS targets, while maintaining a sufficient signal-to-noise ratio and employing short STFT integration windows to increase the measurement resolution of the target’s instantaneous velocity.

We demonstrate this approach for the detection of various types of fired bullet flying in supersonic velocities. It was demonstrated that the moving object’s velocity, as well as its drag constant, can be evaluated by tracking its instantaneous speed during time of flight. Furthermore, a comparison between the experimental drag constant obtained from the radar and the theoretical drag constant was made, which showed a high correlation. Estimating the drag constant can contribute to understanding the geometry and mass of a small supersonic moving target.

This detection technique enables tracking and identifying the sources of gunshots, determining the instantaneous velocity of fast targets, and analyzing the ballistic movement of an object.

Using a radar operating at an extremely high frequency band has advantages over other sensors due to its directivity and high-resolution detection features. The short wavelength facilitates using small aperture antennas and reduces equipment size.
